# Effect of elbow flexion angles on stress distribution of the proximal ulnar and radius bones under a vertical load: measurement using resistance strain gauges

**DOI:** 10.1186/s13018-014-0060-0

**Published:** 2014-07-31

**Authors:** Zhi-Tao Rao, Feng Yuan, Bing Li, Ning Ma

**Affiliations:** 1 Department of Orthopaedics, Tongji Hospital of Tongji University School of Medicine, No. 389 Xincun Road, Shanghai 200065, China

**Keywords:** Elbow joint, Strain, Biomechanics, Resistance strain method

## Abstract

**Objectives:**

This study aimed to explore the surface stress at the proximal ends of the ulna and radius at different elbow flexion angles using the resistance strain method.

**Methods:**

Eight fresh adult cadaveric elbows were tested. The forearms were fixed in a neutral position. Axial load increment experiments were conducted at four different elbow flexion angles (0°, 15°, 30°, and 45°). Surface stain was measured at six sites (tip, middle, and base of the coronoid process; back ulnar notch; olecranon; and anterolateral margin of the radial head).

**Results:**

With the exception of the ulnar olecranon, the load-stress curves at each measurement site showed an approximately linear relationship under the four working conditions studied. At a vertical load of 500 N, the greatest stress occurred at the middle of the coronoid process when the elbow flexion angles were 0° and 15°. When the flexion angles were 30° and 45°, the greatest stress occurred at the base of the coronoid process. The stress on the radial head was higher than those at the measurement sites of the proximal end of the ulna.

**Conclusion:**

The resistance strain method for measuring elbow joint surface stress benefits biomechanics research on the elbow joint. Elbow joint surface stress distributions vary according to different elbow flexion angles.

## Introduction

The elbow joint belongs to a composite joint composed of the humeroulnar joint, humeroradial joint, and proximal radioulnar joint encapsulated by a joint capsule [1]-[3]. The proximal ends of the ulna (coronoid process and ulnar notch) and radius (radial head) are important proximal constituents of the elbow joint. Complex elbow fracture dislocations, such as fracture dislocation of the olecranon, posterior monteggia fracture dislocation, and terrible triad injury of the elbow, are caused by various injuries, most of which involve the proximal bone architectures of the ulna and radius as well as the soft tissue [4]-[7]. The different injury types are generally correlated with the flexion and extension positions of the elbow as well as the rotational position of the forearm when the injury was incurred [8]-[10]. Therefore, understanding the pathomechanisms of elbow injury is essential in the diagnosis and treatment of complex elbow fracture dislocations. The resistance strain measurement technique has attracted increased attention from scholars as a precise strain measurement method in the field of biomechanics. As such, this technique has been extensively applied in experimental stress analysis in recent years [11]-[13].

It is well known that the stresses differ when the elbow is fully extended or locked versus when it is flexed at different angles. However, no experimental measurement has yet quantitatively analyzed the bone surface stress of the elbow. In particular, no study has explored the variation trends of the surface stress at different degrees of flexion. Understanding the change in trends of stress with flexion angles may help clinicians comprehend the pathological mechanisms of special elbow injury types and develop appropriate surgical procedures and proper postoperative rehabilitation strategies. In this study, the surface load stress distributions of the ulna and radius were measured at different elbow flexion angles under a vertical load using the resistance strain measurement technique. The correlation between elbow flexion angles and injury types was also explored.

## Materials and methods

### Subjects

Eight fresh cadaveric upper limb specimens were supplied by the Department of Anatomy and Neurobiology of Tongji University, China. This study was conducted in accordance with the declaration of Helsinki and performed with the approval from the Ethics Committee of Tongji Hospital of Tongji University, School of Medicine. Written informed consent was obtained from all donated families. The subjects (five men, three women) were 57 to 79 years old (mean, 67.75 ± 6.82) and had never incurred upper limb lesions, such as malformations, trauma, or bone diseases. The specimens were stored at −20°C for 1 to 21 days (mean, 8). The specimens were defrosted at room temperature approximately 16 h prior to the experiment. The specimen was cut from the distal part of the humerus (proximal end) 15 cm above the elbow joint to the antebrachiocarpal joint (distal end), and the distal radioulnar joint ligaments were retained. The skin, fascia, and muscles were removed, while the capsule and ligaments around the elbow joint were kept intact.

### Specimen preparation and measurement point determination

**Figure 1:**
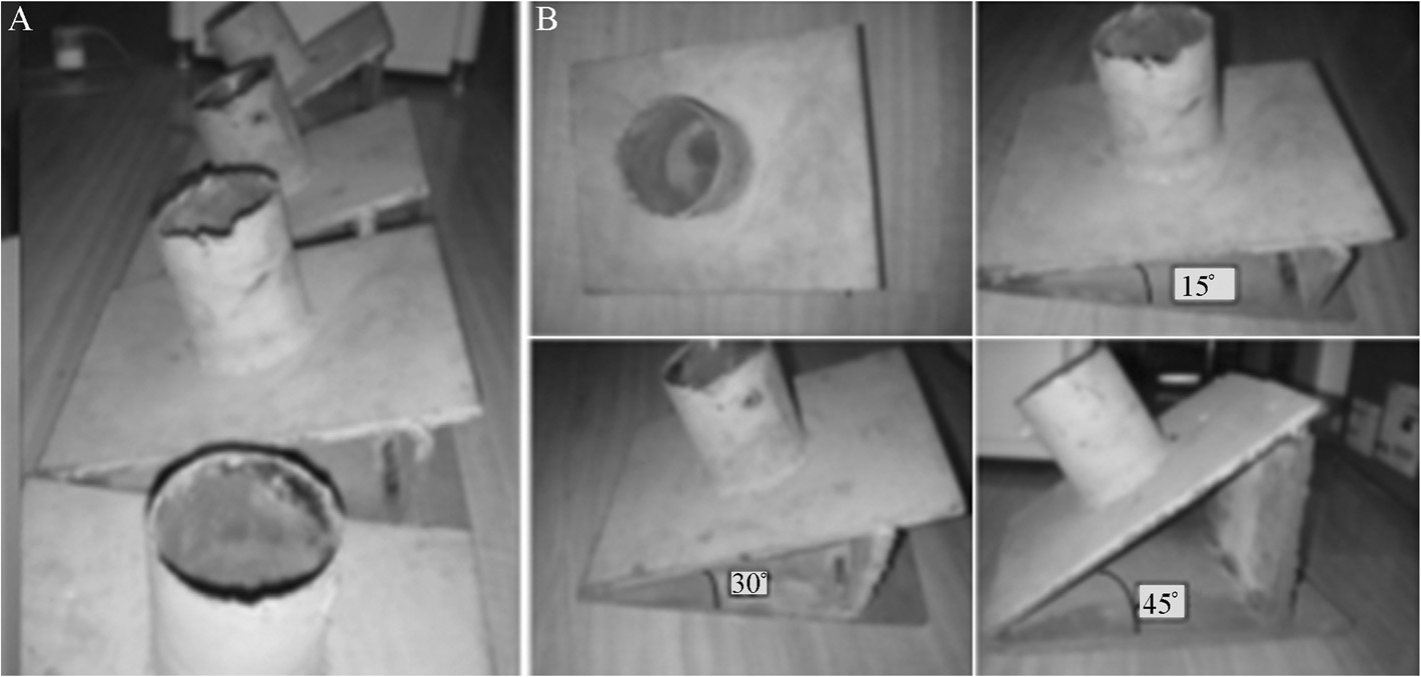
**Special elbow joint specimen fixators (AandB).** When the forearm in a neutral position is fixed vertically in the fixator and the humerus is kept in a vertical position, the fixator angle is equal to the flexion of the elbow joint at 0°, 15°, 30°, and 45°.

**Figure 2:**
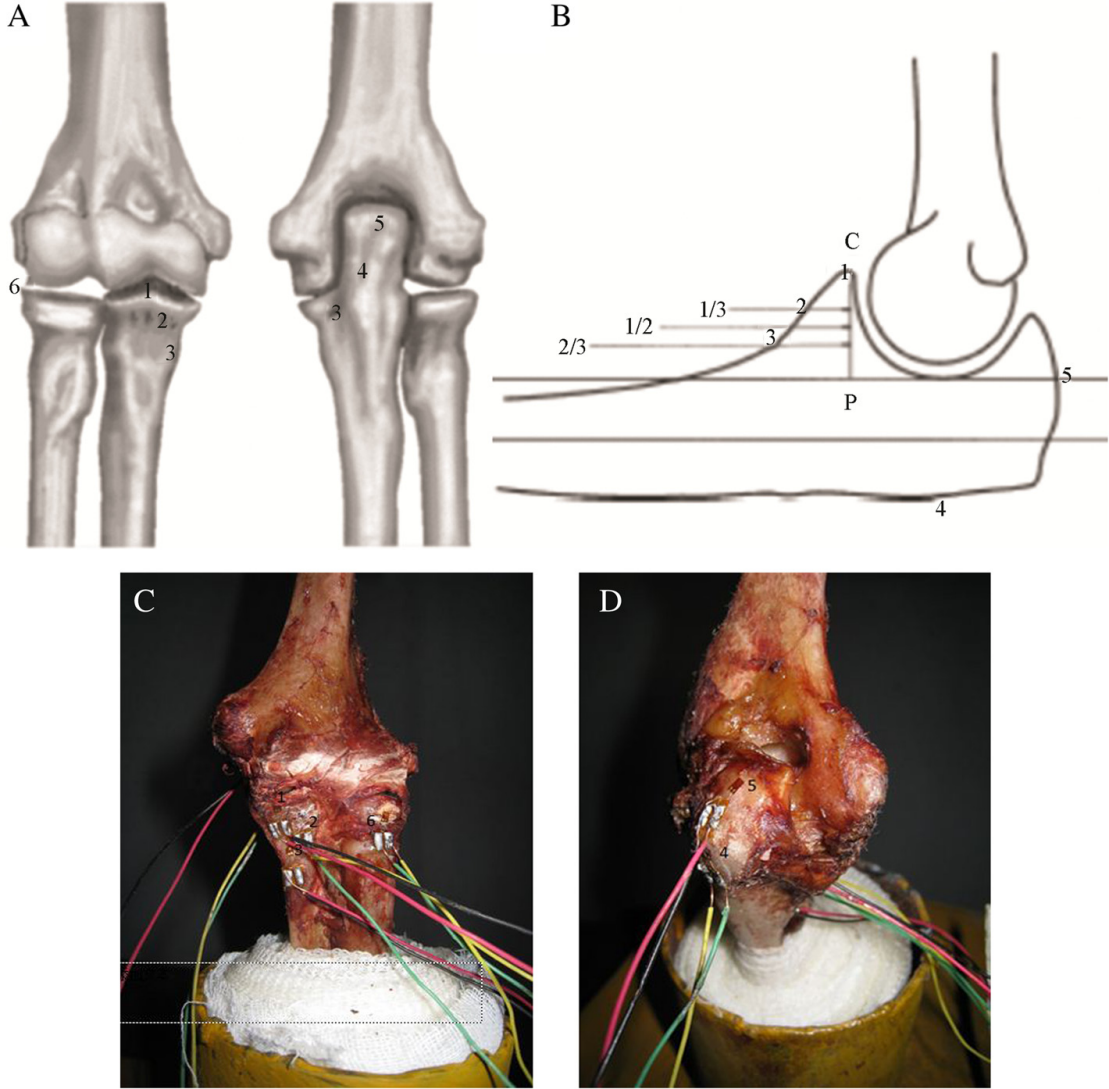
**The elbow measurement sites. (AandB)** Schematic diagrams of the pasting sites of the resistance strain gauges. The distance between points *C* and *P* represents the height of the coronoid process. **(CandD)** The front and dorsal views of elbow specimens and the connection positions of resistance strain gauges, respectively. 1 = the ulna coronoid process tip (located at the ulna coronoid height 1/3, near the tip), 2 = the middle of the coronoid process (the 1/2 height site), 3 = base part of the coronoid process (the 2/3 site of the height, near the base), 4 = the back of ulnar notch, 5 = the olecranon tip, 6 = the anterolateral margin of the radial head.

The distal and proximal ends of the specimens were pre-wrapped using polymer plaster and self-curing denture base acrylic resin powder. The distal ends of the ulna and radius bones were placed in a neutral position and fixed at varying angles (0°, 15°, 30°, and 45°) on a metal frame (Figure 1). Six bone measurement points were marked: tip (within one third of its length), middle (half of its length), and base (two thirds of its length) of the coronoid process; back ulnar notch; olecranon; and anterolateral margin of the radial head (Figure 2). The distance between points C and P in the figure denotes the height of the coronoid process. A 5-mm transverse incision was made on the necessary joint capsule to expose the coronoid process tip of the ulna and the anterolateral margin of the radial head. The coronoid process tip and anterolateral margin of the radial head were exposed, and the longitudinal integrity of the anterior and lateral joint capsule was retained. The anterolateral margin of the radial head was exposed above the annular ligament of the radius, and the integrity of the proximal radioulnar joint was retained. The six measurement points were degreased with acetone and dehydrated with alcohol. After drying, the bone surface was polished using fine sandpaper. A straight line was vertically marked on the surface along the axial walking direction of the trabecular bone with a thin needle that was used as the strain gauge attachment positioning mark.

### Strain gauge welding, pasting, and connecting

The guidewires and connecting leads of the miniature foil resistance strain gauges (BE-120-1AA-W (16); sensitive gate size, 1.0 mm × 2.0 mm; electric resistance, 120.3 ± 0.1 Ω; and sensitivity coefficient, 2.01 ± 0.01; ZEMIC, Beijing, China) were welded to the connection terminals. A thin layer of ethyl cyanoacrylate instant adhesive (502 glue) was applied on the marked sites of the bone surface as well as the required surfaces of the strain gauges and the connection terminals. After ensuring firm adhesion, a multimeter was used to perform short-circuit testing of the connection guidewires. Six strain gauges were used for each specimen. The strain gauges were adhered to the corresponding bone sites according to their serial numbers and then connected to a static strain tester (DH type 3818; DongHua Test, Taizhou, China) through a 1/4 bridge (multichannel sharing compensating gauge). The parameters of sensitivity coefficient, guidewire electric resistance, and strainmeter electric resistance were used as inputs. The digital strainmeters were pre-adjusted (calibration, zero basing, measurement, and attenuation) to ensure a stable resistance strain and avoid null shifts. The attenuation block was set to 10, and the sensitivity coefficients of the tester and resistance gauges were maintained to ensure the consistency of the resistance values of all test pieces.

### Mechanic loading

**Figure 3:**
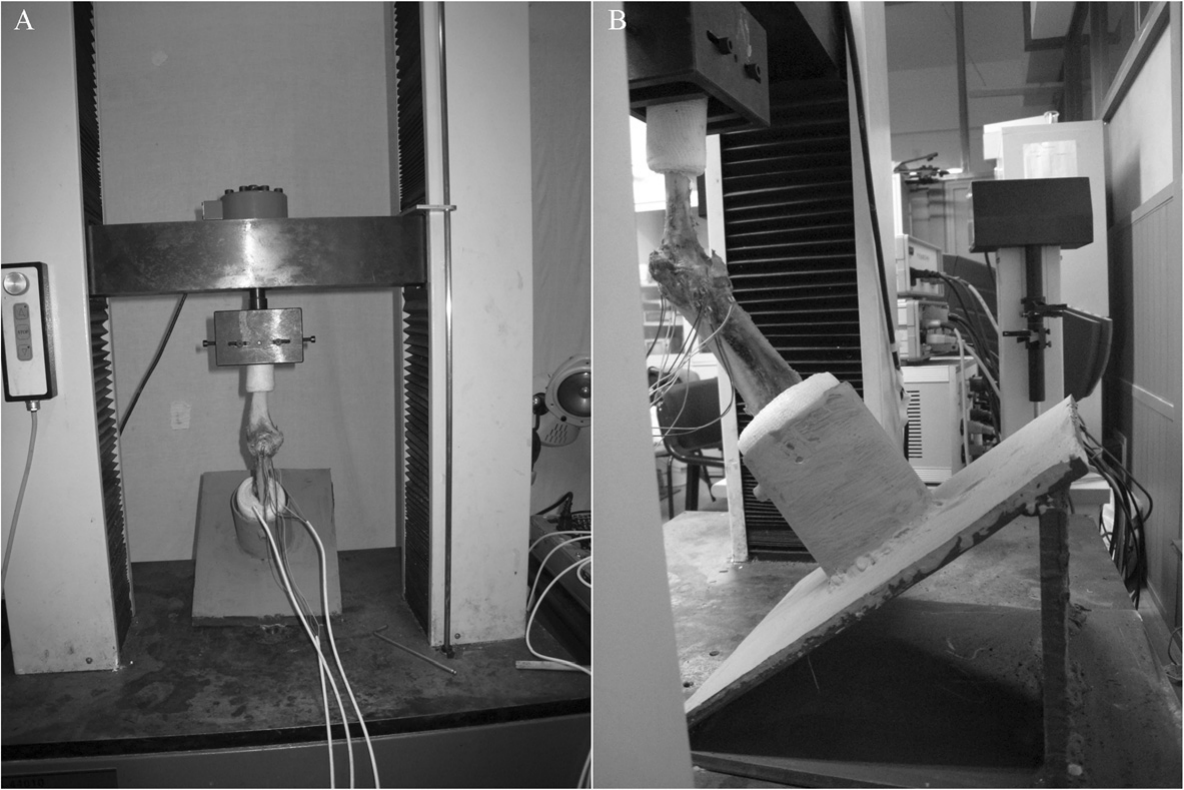
Experimental facility location (A) and mechanic experiment scene (B).

A wedge-shaped specimen frisket was fixed on the substrate of the laboratory table. The distal ends of the specimen were fixed vertically in the socket of the fixator at different degrees, and the proximal ends of the humerus were fixed vertically with the ground to ensure the relative fixation of the flexion angle, the angle between the inclined plane of the frisket and the horizontal plane. Vertical hydraulic loading was performed on the specimens at a rate of 10 mm/min using an electric universal test machine (CSS-44010; KeXin Testing Machine Co., Ltd., Beijing, China; Figure 3). Pre-loading of 100 N was performed before each experiment. The load levels were divided at 100-N intervals to 500 N. Each experiment was repeated three times, and the mean values were used in the analyses.

### Data processing

The microstrain at each measurement site was recorded to calculate the mean and standard deviation under each working condition. The stress was calculated according to the complex stress state theory in the field of materials mechanics provided that the bones were viewed as homogenous, continuous, and isotropic linear elastomeric materials. The correlation between stress and strain was calculated based on Hooke's law, that is, *σ* = *E* · *ε*, where *E* is the constant of elastic modulus, *σ* is the stress value, and *ε* is the strain value measured in the experiment. According to the literature, the elastic modulus of bone architecture is 7.3 GPa [14]. Thus, the stress value at each measurement point was obtained on the basis of this formula. The positive and negative signs prior to the strain value represent the compression and elongation of the strain gauge, which correspond to the compressive or tensile stress at this measurement point, respectively.

### Statistical analysis

Data were analyzed using SPSS 16.0 software. The load-stress correlations preliminarily showed variance homogeneity. Linear regression analysis was performed. One-way analysis of variance (ANOVA) was used to examine the differences in the strains at the different measurement sites and flexion angles. Differences among the groups under different working conditions were also examined with one-way ANOVA. Independent samples *t* tests were used to compare the groups. Differences of *P* < 0.05 were considered statistically significant.

## Results

### Microstrain of the elbow at different flexion angles

No frisket slippage, specimen fractures, or elbow joint dislocations occurred during the experiment. The electric measurements under different working conditions showed that under a vertical load alone, the strain values at the measurement sites of the coronoid process and radial head were negative, suggesting compressive stress. In contrast, the strain values at the measurement sites of the back ulnar notch and olecranon were positive, suggesting tensile stress.

**Figure 4:**
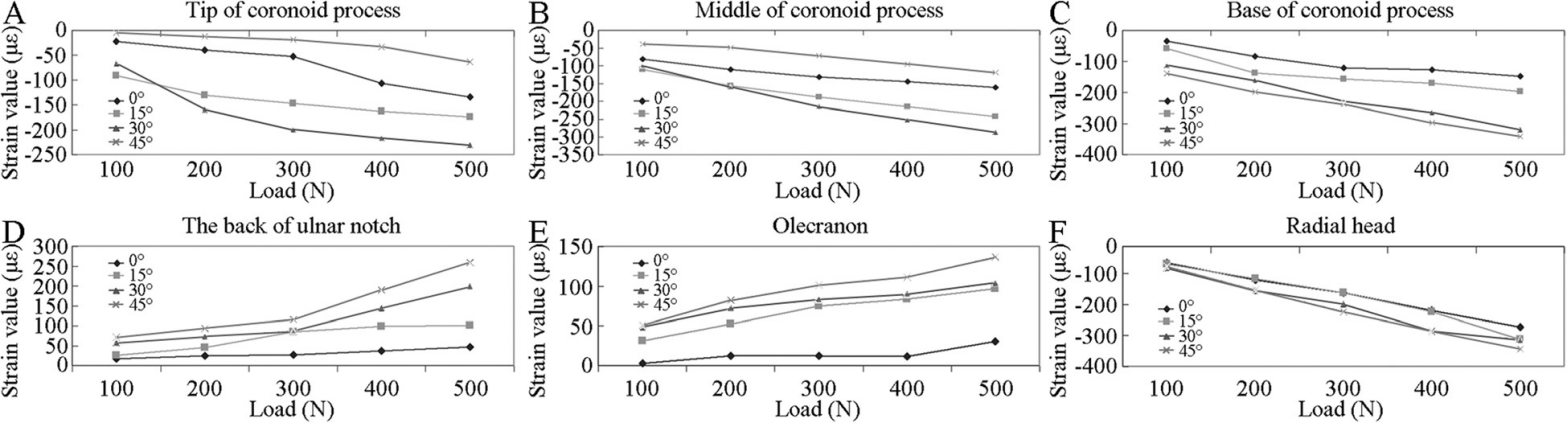
**Load-strain curves of different elbow measurement sites under different conditions of elbow flexion. (A)** Tip of the coronoid process; **(B)** middle of coronoid process; **(C)** base of coronoid process; **(D)** the back of ulnar notch; **(E)** the olecranon; **(F)** the radial head.

The strain values at all measurement points under the different working conditions increased as the vertical load increased (*P* < 0.05). However, no correlation was observed at the olecranon measurement site when the elbow flexion angle was 0° (extension position), which indicates approximately linear relationships (Figure 4A,B,C,D,E,F).

### Strain and stress of the elbow joint under vertical loads

**Figure 5:**
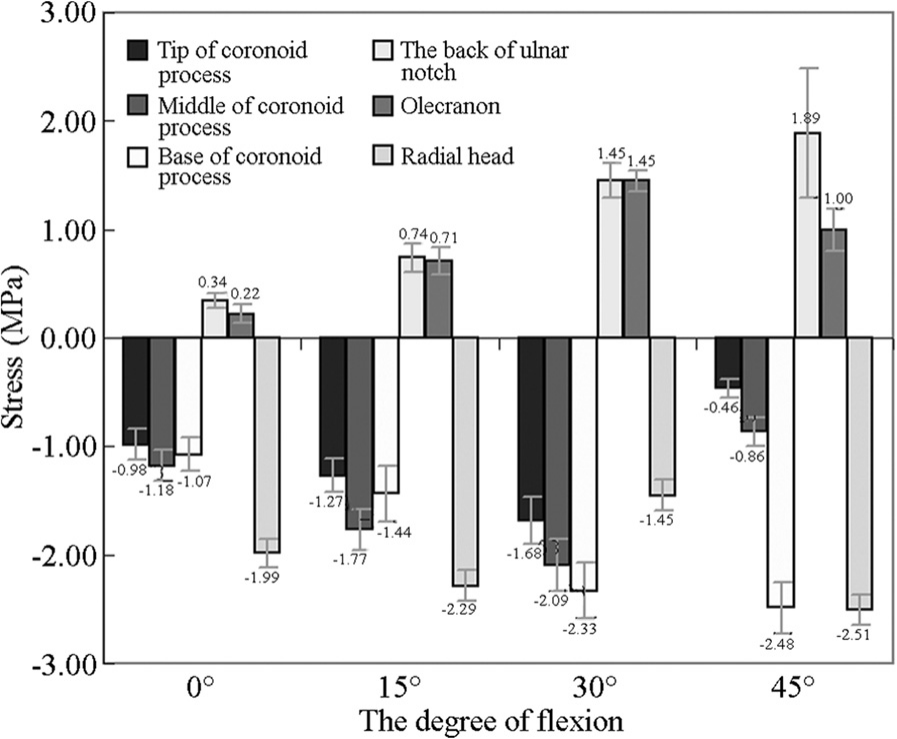
**Stress values (in MPa) of the bone measurement sites at different elbow flexion angles.** The vertical load is 500 N.

For convenience, a vertical load of 500 N was used when the microstrain values at different measurement points were compared (Table 1). The stress value at each measurement point was calculated using Hooke's law (Figure 5). The results of the statistical analysis showed that the strain at each measurement point displayed specific variation trends depending on the flexion angle of the elbow joint. Variations caused by varying flexion angles in the stress at the tip and middle of the coronoid process at a vertical load of 500 N showed identical trends as follows: stress at 30° > stress at 15° > stress at 0° > stress at 45°. At the same flexion angle, the stress at the middle of the coronoid process was significantly greater than that at the tip (*P* < 0.05). At the base of the coronoid process, high stress values occurred when the flexion angles were 45° and 30° (*P* = 0.19), followed by 15° and 0° (*P* < 0.05).

**Table 1:** Stress values at different elbow flexion angles under a vertical load of 500 N (in με; mean ± SD)

**Working condition**	**Tip of coronoid process**	**Middle of coronoid process**	**Base of coronoid process**	**The back of ulnar notch**	**Olecranon**	**Radial head**
0°	−134.38 ± 19.93	−161.38 ± 19.76	−147.25 ± 21.01	46.75 ± 9.50	30.38 ± 12.21	−272.13 ± 17.52
15°	−173.38 ± 21.46	−242.25 ± 26.54	−196.75 ± 34.94	101.63 ± 17.80	97.13 ± 17.32	−313.38 ± 19.98
30°	−230.75 ± 29.52	−286.50 ± 32.82	−319.50 ± 34.88	198.63 ± 21.89	104.13 ± 13.89	−313.00 ± 19.93
45°	−63.25 ± 11.97	−118.25 ± 17.59	−340.38 ± 32.16	259.38 ± 81.49	136.63 ± 27.09	−343.75 ± 19.17
*P*	<0.05*	<0.05*	>0.05	<0.05*	>0.05	>0.05

^*^P < 0.05 indicates a statistically significant difference.

At the back measurement site of ulnar notch, the flexion angles of the elbow joint can be arranged from the highest to the lowest stress values as follows: 45° > 30° > 15° > 0°; all variations showed significant differences (*P* < 0.05). Variations in stress at the olecranon measurement point were also compared at the different working conditions of 15°, 30°, and 45°. The stress value at 45° was noticeably greater than those at 15° and 30° (*P* < 0.05), while no significant difference was observed between the two latter working conditions (*P* = 0.496).

At a vertical load of 500 N, the stress values at the measurement site of the radial head were high. The maximum and minimum values appeared at 45° and 0°, respectively, and showed a significant difference (*P* < 0.05). However, no significant difference was observed between the values at 15° and 30° (*P* = 0.97).

## Discussion

In this study, the load-strain curves of all the measurement sites of the proximal ends of the ulna and radius showed approximately linear relationships with the exception of the olecranon measurement point at 0° flexion. When the elbow is fully extended, the stress under the vertical load is mainly distributed on the front of the humeroulnar joint. As the elbow flexion angle increases, the stress distribution of the humeroulnar joint becomes gradually concentrated on the rear of the olecranon. Moreover, bones are elastomeric materials. When the load on the joint exceeds the ultimate limit, fractures can occur. Wake et al. [9] carried out a biomechanical study of the mechanisms of elbow fracture dislocation caused by vertical compressive violence and found that injury type had a definite correlation with elbow flexion degree. When the elbow was at 60° or 90° flexion, an olecranon fracture and anterior elbow dislocation might occur. When the elbow flexion angle is between 30° and 15°, a coronoid process fracture or posterior elbow dislocation might happen. Additionally, the coronoid process fragment size was associated with the degree of elbow extension, that is, a larger extension angle indicates smaller fragments.

The results of the present study show that the stress on the proximal ulna is focused on the middle of the coronoid process height when the elbow is at 0° or 15° flexion under a vertical load and focused on the base of the coronoid process when the elbow is at 30° or 45° flexion. This finding suggests that when the elbow flexion is 0° to 15°, injury may cause middle coronoid process fractures, the equivalent of Regan-Morrey type II fractures. At flexion of 30° to 45°, injury may cause Regan-Morrey type III fractures [15]. As the elbow flexion increases, the stress concentration gradually decreases. These findings are consistent with the report by Doornberg et al. [16] on complex elbow fractures such as monteggia fractures and olecranon fracture dislocation, both of which are manifested by olecranon fractures complicated by large coronoid process fragments.

The special stress concentration positions at different flexion angles under a vertical load suggest that coronoid process fragment size may be correlated with elbow flexion degree. In this study, no stress was concentrated on the tip of the coronoid process under the elbow flexion angles of 0°, 15°, 30°, and 45°. However, coronoid process tip fractures account for a large proportion of cases in clinical practice, and most are accompanied by posterior elbow dislocation. Anatomical studies found that coronoid process tip fractures were not subject to avulsion fracture [17],[18]. Most coronoid process tip fractures presumably occur with full elbow extension and posterior elbow dislocation. During full extension, the stress enables the coronoid process tip to contact and collide with the humerus trochlea, resulting in fractures.

The current experiment revealed that the surface stress of the measured radial head point is higher than that of the proximal ulna when the elbow flexion angle was 0° to 45°, suggesting that the anterolateral margin of the radial head endures greater axial stress than the proximal ulna. Since the elbow joint has a certain valgus angle (carrying angle), 60 % of the vertical load is concentrated on the humeroradial joint [2],[3]. This finding is consistent with the observation in clinical practice that coronoid process fractures alone are rare among severe elbow joint injuries, which are most frequently combined with radial head fractures [19].

This study provides novel insights into the mechanisms underlying the different types of complex elbow fracture dislocations using the resistance strain method; however, it has many limitations. First, only eight cadaveric elbow specimens were used, all of which were from elderly patients (57 to 79 years old), while peri-joint soft tissue injuries (e.g., collateral ligaments, joint capsule, extensor and flexor tendon attachments, and triceps attachments of the olecranon) were not explored despite the fact that they cause elbow instability after bone structure reconstruction [5],[7],[20],[21]. Second, this study adopted the load increment method, which cannot accurately reflect the actual injury; that is, the static load loading biomechanical study cannot stimulate the dynamic changes that occur during a fall. Third, the forearms were placed in a neutral position, whereas prone or supine forearm rotation may occur during injury. Fourth, considering that the elbow has a certain carrying angle, violence can either be induced by vertical, lateral, rotational, or combined loads. Finally, the study analyzed the surface stress of the bone structure using the resistance strain gauge measurement method, which has high sensitivity and accuracy but can measure only bone surface stress. As such, the pressure distribution inside the joints remains to be studied, and the damage process requires further investigations.

## Conclusion

The joint surface stress can be measured using resistance strain gauges. The measurement results provide novel insights into biomechanics research on the elbow joint. The surface stresses of the proximal ulna and radius bone distributions vary among different elbow flexion angles under a vertical load. Further studies determining factors that improve outcome are warranted.

## Competing interests

The authors declare that they have no competing interests.

## Authors’ contributions

ZTR participated in the design of the study, performed the data collection, and drafted the manuscript. FY initiated the study, participated in the design of the study, revised the manuscript critically for important intellectual content, and has given final approval of the version to be published. BL and NM participated in the design of the study and performed the statistical analysis. All authors read and approved the final manuscript.

## References

[B1] Heim U Kombinierte verletzungen von radius and ulnar in proximalen unteram segment Hefte Unfallechir 1994 241 61 79 10.1007/978-3-662-00855-3_11

[B2] Fomalski S Gupta R Lee TQ Anatomy and biomechanics of the elbow joint Tech Hand Upper Extremity Surg 2003 7 4 168 178 10.1097/00130911-200312000-00008 16518218

[B3] Bryce CD Armstrong AD Anatomy and biomechanics of the elbow Orthop Clin North Am 2008 39 2 141 154 10.1016/j.ocl.2007.12.001 18374805

[B4] Pugh DM Wild LM Schemitsch EH King GJ McKee MD Standard surgical protocol to treat elbow dislocations with radial head and coronoid fractures J Bone Joint Surg Am 2004 86A 6 1122 1130 15173283 10.2106/00004623-200406000-00002

[B5] Bell S Elbow instability, mechanism and management Curr Orthopaed 2008 22 90 103 10.1016/j.cuor.2008.04.007

[B6] Harrison JW Chitre A Lammin K Warner JG Hodgson SP Radial head fractures in adults Curr Orthopaed 2007 21 1 59 64 10.1016/j.cuor.2006.10.003

[B7] Giannicola G Sacchetti FM Greco A Cinotti G Postacchini F Management of complex elbow instability Musculoskelet Surg 2010 94 1 S25 S36 10.1007/s12306-010-0065-8 20383679

[B8] Doornberg JN Ring D Coronoid fracture patterns J Hand Surg Am 2006 31 1 45 52 10.1016/j.jhsa.2005.08.014 16443103

[B9] Wake H Hashizume H Nishida K Inoue H Nagayama N Biomechanical analysis of the mechanism of elbow fracture-dislocations by compression force J Orthop Sci 2004 9 1 44 50 10.1007/s00776-003-0735-6 14767704

[B10] Gradl G Jupiter JB Current concepts review—fractures in the region of the elbow Acta Chir Orthop Traumatol Cech 2012 79 3 203 212 22840951

[B11] Eser A Akça K Eckert S Cehreli MC Nonlinear finite element analysis versus ex vivo strain gauge measurements on immediately loaded implants Int J Oral Maxillofac Implants 2009 24 3 439 446 19587865

[B12] Fresvig T Ludvigsen P Steen H Reikerås O Fibre optic Bragg grating sensors: an alternative method to strain gauges for measuring deformation in bone Med Eng Phys 2008 30 1 104 108 10.1016/j.medengphy.2007.01.006 17369073

[B13] Funk JR Crandall JR Calculation of tibial loading using strain gauges Biomed Sci Instrum 2006 42 160 165 16817602

[B14] Choi K Kuhn JL Ciarelli MJ Goldstein SA The elastic moduli of human subchondral, trabecular, and cortical bone tissue and the size-dependency of cortical bone modulus J Biomech 1990 23 11 1103 1113 10.1016/0021-9290(90)90003-L 2277045

[B15] Regan W Morrey BF Fractures of the coronoid process of the ulna J Bone Joint Surg 1989 71 9 1348 1354 2793888

[B16] Doornberg JN van Duijn J Ring D Coronoid fracture height in terrible-triad injuries J Hand Surg Am 2006 31 5 794 797 10.1016/j.jhsa.2006.01.004 16713844

[B17] Cage DJ Abrams RA Callahan JJ Botte MJ Soft tissue attachments of the ulnar coronoid p rocess. An anatomic study with radiographic correlation Clin Orthop 1995 320 154 158 7586820

[B18] Ablove RH Moy OJ Howard C Peimer CA S'Doia S Ulnar coronoid process anatomy: possible implications for elbow instability Clin Orthop 2006 449 259 261 16672900 10.1097/01.blo.0000218729.59838.bc

[B19] Kälicke T Muhr G Frangen TM Dislocation of the elbow with fractures of the coronoid process and radial head Arch Orthop Trauma Surg 2007 127 10 925 931 10.1007/s00402-007-0424-6 17713772

[B20] Sørensen AK Søjbjerg JO Treatment of persistent instability after posterior fracture-dislocation of the elbow: restoring stability and mobility by internal fixation and hinged external fixation J Shoulder Elbow Surg 2011 20 8 1300 1309 10.1016/j.jse.2011.06.002 21982348

[B21] Jupiter JB Baptista CM Simultaneous reconstruction of both medial and lateral collateral ligament complexes for recurrent instability of elbow dislocation: a case report Surg Orthop Adv 2012 21 4 266 269 10.3113/JSOA.2012.0266 23327854

